# Advances in Simple, Rapid, and Contamination-Free Instantaneous Nucleic Acid Devices for Pathogen Detection

**DOI:** 10.3390/bios13070732

**Published:** 2023-07-14

**Authors:** Yue Wang, Chengming Wang, Zepeng Zhou, Jiajia Si, Song Li, Yezhan Zeng, Yan Deng, Zhu Chen

**Affiliations:** 1 Hunan Key Laboratory of Biomedical Nanomaterials and Devices, Hunan University of Technology, Zhuzhou 412007, China; yuesir0029@163.com (Y.W.); 18834563351@163.com (Z.Z.); sjj975895902@163.com (J.S.); sosong1980@gmail.com (S.L.); 2 Department of Cardiovascular Medicine, The Affiliated Zhuzhou Hospital Xiangya Medical College, Central South University, Zhuzhou 412000, China; 18670232436@163.com; 3 School of Electrical and Information Engineering, Hunan University of Technology, Zhuzhou 412007, China; zengyezhan@126.com

**Keywords:** pathogens, nucleic acid detection, noncontaminating, simple and rapid, POCT

## Abstract

Pathogenic pathogens invade the human body through various pathways, causing damage to host cells, tissues, and their functions, ultimately leading to the development of diseases and posing a threat to human health. The rapid and accurate detection of pathogenic pathogens in humans is crucial and pressing. Nucleic acid detection offers advantages such as higher sensitivity, accuracy, and specificity compared to antibody and antigen detection methods. However, conventional nucleic acid testing is time-consuming, labor-intensive, and requires sophisticated equipment and specialized medical personnel. Therefore, this review focuses on advanced nucleic acid testing systems that aim to address the issues of testing time, portability, degree of automation, and cross-contamination. These systems include extraction-free rapid nucleic acid testing, fully automated extraction, amplification, and detection, as well as fully enclosed testing and commercial nucleic acid testing equipment. Additionally, the biochemical methods used for extraction, amplification, and detection in nucleic acid testing are briefly described. We hope that this review will inspire further research and the development of more suitable extraction-free reagents and fully automated testing devices for rapid, point-of-care diagnostics.

## 1. Introduction

Pathogenic agents are microorganisms that can cause diseases, such as bacteria, viruses, and fungi. These pathogens can either directly affect the host through their virulent effects or indirectly by triggering an immune response and inflammation. Some pathogens produce substances such as cytotoxins, which can destroy host cells and tissues, or elicit adverse reactions in the host’s immune system. Others invade host tissues, leading to tissue necrosis. These pathogens have the potential to negatively impact human health. Some common pathogenic agents include the hepatitis B virus [1], human cytomegalovirus, Nipah virus, novel coronavirus, and various common household germs [2,3,4,5]. Chronic infection with the hepatitis B virus can ultimately result in death due to cirrhosis or hepatocellular carcinoma [6,7]. Human cytomegalovirus is a beta herpesvirus that poses health risks for immunocompromised individuals and may cause mononucleosis. It has also been associated with the development of certain cancers and chronic inflammatory diseases, such as cardiovascular diseases [8]. Nipah virus is an emerging zoonotic virus with multiple outbreaks and high mortality rates. It can lead to encephalitis, systemic vasculitis, and respiratory diseases [9]. The novel coronavirus is a new strain of coronavirus that had not been previously identified in humans [10]. Mild symptoms include loss of taste and smell, gastrointestinal symptoms, and headaches. In severe cases, it can progress to acute respiratory distress syndrome, necessitating ventilatory support [11]. Clostridium difficile infection (CDI) is a significant cause of nosocomial enteric disease, resulting in substantial morbidity and mortality among hospitalized patients worldwide [12]. Streptococcus pneumoniae, also known as “pneumococcus”, is a major cause of both morbidity and mortality globally. It can lead to life-threatening conditions such as pneumonia, bacteremia, and meningitis, with an annual mortality burden exceeding one million cases [13]. Both neocoronaviruses and other pathogenic agents are highly contagious and can be transmitted from human to human. The primary modes of transmission include airborne droplets and close contact. In order to effectively control the development and spread of the epidemic, various detection methods have been developed based on the characteristics of the pathogens. These methods involve detecting antigens and antibodies on the surface of the pathogen, as well as nucleic acids. Depending on the characteristics of the pathogens, different tests have been developed, such as nucleic acid tests [14,15,16], antigen tests [17,18,19], antibody tests [20] and electrochemical sensors [21,22,23], and other aspects [24,25,26]. Nucleic acid testing involves exponentially amplifying the nucleic acid sequence of the pathogen and then using probes or fluorescent markers for detection. Antigen testing, on the other hand, detects the presence or absence of the pathogen by detecting its proteins. Antibody testing involves collecting samples, such as blood, to detect the presence of specific antibodies that bind to the pathogen, indicating the presence of an infection. Although there are several methods for pathogen detection, nucleic acid testing is currently considered the most effective method for detecting novel coronavirus infections and is regarded as the “gold standard” for identifying such infections [27]. However, this method often requires specialized equipment and trained technicians to perform tests in a laboratory setting. Therefore, efforts have been made to develop rapid, portable, and point-of-care devices that can provide nucleic acid test results. Many researchers are currently focusing on developing all-in-one nucleic acid testing devices that are faster, smaller, and more automated [28,29,30,31,32,33]. 

Nucleic acid testing consists of three main steps: nucleic acid extraction [34,35,36,37,38,39,40,41,42,43,44], nucleic acid amplification [45,46,47,48,49,50,51,52,53,54,55,56,57], and detection methods [58,59,60,61,62]. The general procedure for nucleic acid extraction involves collecting the sample to be tested (such as oropharyngeal swabs, fingertip blood, or saliva) and then obtaining the nucleic acid through a process of lysis, separation, precipitation, and purification [63,64,65]. Although direct extraction by high-temperature lysis and lysis buffer can be used, the nucleic acids obtained through this method may be impure and contain contaminants. To enhance the purity of nucleic acid extraction, purification methods such as magnetic bead extraction [66] and centrifugal column purification are employed. Additionally, a nucleic-acid-free extraction method has been developed to reduce the overall testing time [67,68]. By using sample release agents, the amount of nucleic acid extraction is reduced, simplifying the nucleic acid testing process, shortening the testing time, improving detection rates, and making the procedure simple and cost-effective.

**Figure 1:**
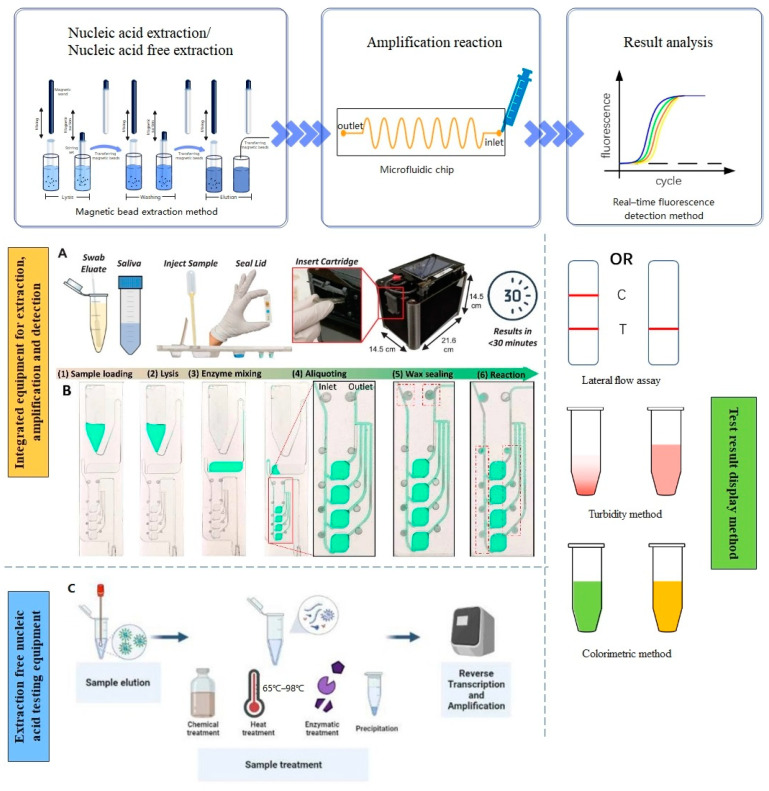
General process of nucleic acid testing: Integrated equipment for extraction, amplification, and detection. (**A**) Reproduced with permission [88]; (**B**) Reproduced with permission [89]; Extraction free nucleic acid testing equipment. (**C**) Reproduced with permission [90].

Nucleic acid amplification is performed exponentially in order to maximize the amount of nucleic acid and achieve significant detection results. There are two types of nucleic acid amplification: variable temperature amplification and isothermal amplification. Variable temperature amplification, such as the PCR [69,70,71,72], consists of three steps: denaturation, annealing, and extension, each carried out at a different temperature. Isothermal amplification, on the other hand, involves adding primers, template, polymerase, and nucleotides to the reaction at a constant temperature, eliminating the need for a thermal cycling system. To facilitate the easy differentiation of the results, various detection methods are used, either visually or indirectly through instruments such as portable fluorescence detectors. Examples of detection methods include real-time fluorescence detection [73], lateral flow detection [74,75,76], visual inspection [77,78], and CRISPR/Cas detection [79,80]. Fluorescence detection involves exposing the fluorescent material in the system to a specific wavelength of light, causing it to emit fluorescence. This fluorescence is then captured by a camera and further analyzed using image processing techniques. Lateral flow assays use immunochromatography principles to detect the presence of a target in a sample, providing qualitative results within minutes. The test sample flows laterally through a matrix via capillary action, allowing for the binding of the antigen to the antibody for detection and visualization purposes. CRISPR/Cas assays, on the other hand, activate the nuclease activity of Cas when a target gene is present in the reaction system. This allows for the degradation of the labeled probe and enables the detection of the target nucleic acid. This assay enables the rapid detection of minute quantities of RNA [81], including influenza virus, HIV virus, or any other RNA viral sequence integrated into the CRISPR enzyme. It offers a fast, sensitive, and straightforward means of RNA detection. The use of lateral flow detection and visual inspection eliminates the need for specialized operators or equipment. When combined with a smartphone, the false positive rate is mitigated to some extent. Artificial intelligence helps scientists in gaining better insights into pathogen aspects, such as biology, genome structure, and transmission pathways, by analyzing vast amounts of data. Machine learning algorithms can predict the spread trends of large-scale outbreaks of specific pathogens, enabling prevention and control measures. Additionally, artificial intelligence coupled with image processing facilitates the rapid and accurate diagnosis of pathogenic infections. As pathogens exhibit variability, AI can identify specific variations and enable targeted interventions. For instance, machine learning or deep learning models can be utilized to enhance accuracy without the need for RNA extraction and pathogen amplification. Smartphones are then incorporated to provide user-friendly interfaces for automated operation. Tripathy et al. developed hybridization reactions without the reverse-transcription polymerase chain reaction, resulting in rapid detection within 30 min for the early diagnosis of novel coronaviruses [82]. Artificial intelligence can enhance the diagnostic efficiency through detailed imaging analysis [83,84]. Walsh et al. successfully employed gradient boosting trees in machine learning to predict influenza a virus in wild birds, demonstrating the high predictive power by detecting various features [85]. Karunakaran et al. devised a label-free surface-enhanced Raman scattering screening method that could differentiate between different coronavirus spike proteins, leveraging machine learning algorithms for the efficient analysis of Raman spectroscopy data [86]. Kokabi et al. demonstrated the ability of neural networks to predict DNA concentrations using raw impedance data, employing a novel machine learning approach for accurate and high-throughput DNA quantification [87]. The general flow of nucleic acid testing is illustrated in Figure 1.

In this review, we will primarily examine the current state of simple, rapid, all-in-one nucleic acid detection systems. These include extraction-free rapid nucleic acid detection, fully automated systems encompassing extraction, amplification, and detection, as well as fully contained and commercially available nucleic acid detection devices. We will also address the challenges associated with these rapid, integrated nucleic acid testing systems and provide an outlook on their future.

## 2. Extraction-Free Rapid Nucleic Acid Testing

Rapid extraction-free nucleic acid detection eliminates the need for nucleic acid extraction and relies on amplification reactions to achieve quick detection. These amplification reactions can be categorized into two main types: thermal cycling, such as the PCR, and isothermal amplification. Thermal cycling can further be classified into spatially based thermal cycling and time-based thermal cycling [91]. Isothermal amplification utilizes various enzymes to facilitate amplification. Both thermal cycling and isothermal amplification methods expedite nucleic acid amplification, enabling ultrafast nucleic acid detection. Current nucleic acid assays for pathogens commonly seen in studies using the two amplification methods described above are shown in Table 1.

### 2.1. Thermal Cycling

Real-time fluorescence (RT-PCR) is considered the gold standard for nucleic acid detection [103]. However, this method has limitations, such as the need for complex testing equipment and specialized personnel to operate it. Additionally, its long detection time makes it unsuitable for point-of-care applications. Therefore, the principles of PCR are applied in microfluidic assays [104,105,106,107]. Thermal cycling can be achieved using two typical methods: spatially based and time-based thermal cycling.

#### 2.1.1. Spatial-Domain-Based Thermal Cycling

Spatial-domain-based thermal cycling involves heating the sample at various temperature intervals to achieve the desired denaturation, annealing, and extension temperatures essential for nucleic acid amplification. This technique includes several approaches such as continuous-flow PCR [108,109,110,111,112,113,114], convective PCR [115,116,117,118,119], oscillatory PCR [120], and spatial-conversion PCR.

**Figure 2:**
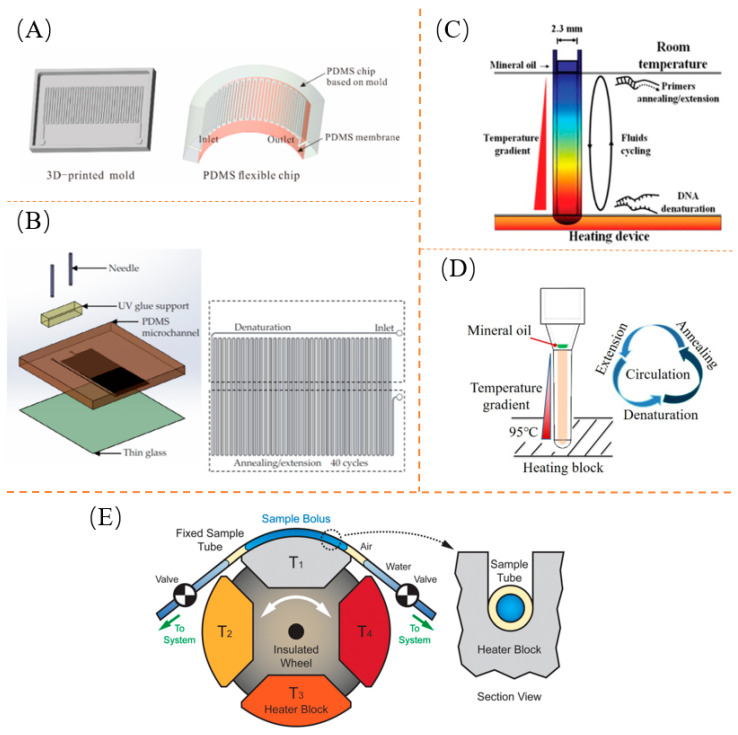
Classification of spatial-domain thermal cycles: (**A**) Continuous-flow PCR. Reproduced with permission [124]; (**B**) Continuous-flow PCR. Reproduced with permission [125]; (**C**) Convective PCR. Reproduced with permission [129]; (**D**) Convective PCR. Reproduced with permission [130]; (**E**) Rotational PCR. Reproduced with permission [133].

Continuous-flow PCR is performed using an elongated channel that is pushed into the reaction chamber at different temperatures through the action of an external pump. This approach ensures reliable temperature control and prevents failure to reach the desired temperature, which could lead to the failure of the entire amplification process. The longer channel increases the surface-area-to-volume ratio, allowing for an efficient interaction between the sample enzymes and the desired temperature. Li et al. investigated the impact of the cross-section, width-to-depth ratio, and length ratio of the three temperature zones within the microchannel for detecting Dengue mimic spirochetes. They discovered that the fluid flux was maximized when the width-to-height ratio was 1:1. Based on this ratio, a portable automated system was designed [121]. However, some continuous-flow PCR devices are bulky and not suitable for portability. Additionally, they require costly external pumps for sample injection. To address these issues, Li et al. developed a portable all-in-one microfluidic device that integrates the CF-PCR and an electrophoresis biochip [122]. This innovative design eliminates the need for expensive external pumps by implementing an automated sample injection directly into the reaction chamber, enabling in situ pathogen detection in the field. Another noteworthy advancement by Wu et al. involved the development of a self-activating pumping mechanism [123]. They utilized end-open impermeable quartz capillaries for passive delivery. Furthermore, Wang et al. utilized 3D printing and magnetic silica beads for efficient target DNA extraction from numerous bacterial samples. They combined this technique with microfluidic PCR to detect bacteria [124], using a microfluidic chip shown in Figure 2A. The microfluidic chip used in this study achieved a DNA extraction efficiency of over 90%, with a lower limit of detection for Salmonella at 102 CFU/mL. Lin et al. explored continuous flow PCR for ultrafast DNA amplification [125]. Their chip design featured a channel height of 56 µm and a thermal cycle length of 4 cm, enabling the completion of 40 thermal cycles within 160 s. The chip design is shown in Figure 2B. Shi et al. presented a handheld real-time fluorescent qPCR system for the quantitative analysis of nucleic acid molecules using a PVC microreactor and a real-time quantitative PCR assay system that can detect simulated H7N9 avian influenza genomic DNA across a concentration range of four orders of magnitude [126]. 

Convective PCR is achieved by inducing thermal convection within the capillary. It is a phenomenon in which the temperature of the upper and lower surfaces is maintained constantly in an enclosed space, resulting in the upward movement of hot fluid and the downward movement of cold fluid. This technique is applied to nucleic acid amplification, allowing spatially separated denaturation, annealing, and extension processes to occur due to temperature gradients. Multiple bacteria DNA targets can be quantified quickly and sensitively by single-nucleotide discrimination in the annular convection chamber by Khodakov et al. [127]. The annular PCR system takes 15 min to reach Ct = 25, which is three times faster than conventional qPCR instruments (45 min), and therefore offers the advantage of a portable device for the rapid and highly multiplexed detection of nucleic acids in immediate diagnostics. A stand-alone convective PCR (cPCR) device connected to a smartphone was developed by Rajendran et al. [128] using a customized heat block controlled by Bluetooth wireless communication to amplify multiple DNA samples simultaneously, allowing target nucleic acids to be amplified in less than 30 min. Chou et al. used capillary convection PCR (CCPR) to heat the bottom of the capillary at 95 °C to allow samples to pass through for CCPCR amplification [129]. The end result was the successful amplification of DNA sequences from three different viral genomes within 30 min. The principle of how device-convective PCR works is shown in Figure 2C. A microfluidic chip for automated nucleic acid extraction and a handheld real-time CPCR system for rapid amplification were developed by Xu et al. [130]. The amplification time has been significantly reduced to 30 min using CPCR compared to the conventional PCR. This demonstrates that reasonable concentrations down to 1.0 TCID_50_/mL of simian influenza A (H1N1) virus can be successfully detected by microfluidic systems, as shown in Figure 2D.

Oscillatory-flow PCR combines the advantages of static PCR and flow-through PCR by allowing sample reaction droplets to move back and forth between different temperature zones. Kopparthy et al. developed a versatile system based on the oscillatory-flow method used in thermal gradient systems for nucleic acid analysis [131]. They simply sandwiched double-sided adhesive Kapton tape and polydimethylsiloxane spacers between glass microscope slides using rapid prototyping to fabricate the microfluidic device. This system was used for the detection of simulated viral phage DNA. A direct comparison between the oscillatory-flow system and commercial PCR instruments showed complete agreement in PCR data and reduced sample-to-result times. 

Spatial-conversion PCR utilizes a rotary/spiral drive to achieve rapid temperature rise and fall, as well as temperature cycling, in fluorescent PCR technology. It fixes the desired temperature module and rotates the microfluidic chip carrying the sample to be tested to the desired temperature, thereby amplifying it. For instance, Jung et al. proposed a rotating PCR gene analyzer [132] that combined a heat block and resistance temperature detector (RTD) for thermal cycling control. They also used a disposable PCR microchip and a stepper motor for the highly sensitive and rapid identification of viral RNA. They achieved this with high sensitivity and speed in identifying influenza virus RNA, mimicking H3N2, H5N1, and H1N1. Alternatively, Bartsch et al. proposed a rotating zone thermal cycler, which is a novel wheel-based device [133]. It has the capability to cycle up to four different fixed temperature blocks in contact with a fixed 4 µL capillary-bound sample, achieving a second conversion of 1–3 steady-state heater powers of less than 10 W. The test sample in this case is a plasmid with a specific sequence. A diagram showing the operation of the rotary PCR is depicted in Figure 2E. The system has a nominal warming rate of up to 44 °C/s and an average time required for cyclic amplification of 21 min. Furthermore, Li et al. developed a fully automated whole-blood hepatitis B virus rapid assay system based on a dual rotary axis centrifugal microfluidic platform (DRA-CMP) [134]. They successfully performed a sample-to-answer assay for HBV using a 500 µL whole-blood sample. The total time from sample to result for the entire assay was approximately 48 min.

#### 2.1.2. Time-Domain-Based Thermal Cycling

Time-domain thermal cycling involves keeping the sample stationary during temperature changes. It includes contact heating [135,136,137,138,139,140,141], as well as noncontact heating methods such as infrared heating [142], laser heating [143] and microwave heating [144].

**Figure 3:**
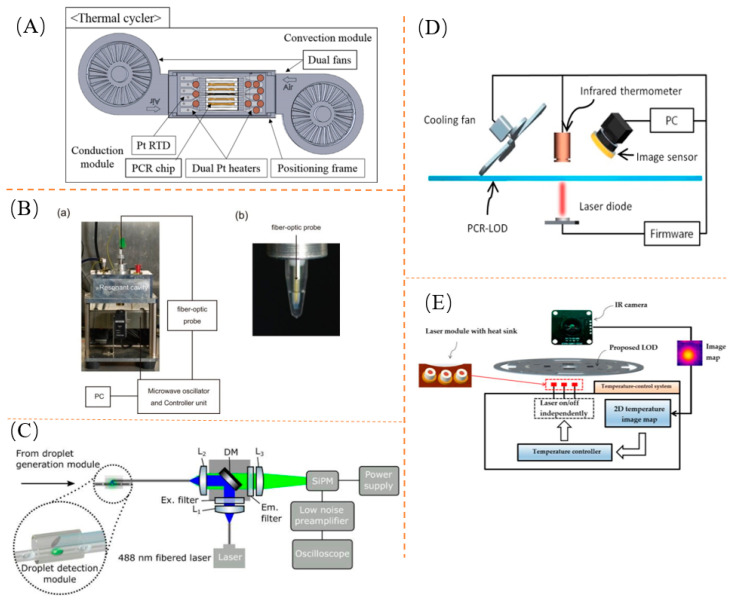
Time-domain thermal cycling: (**A**) Contact heating. Reproduced with permission [148]; (**B**) Microwave heating. Reproduced with permission [157]; (**C**) Microwave heating. Reproduced with permission [158]; (**D**) Infrared laser heating. Reproduced with permission [159]; (**E**) Infrared laser heating. Reproduced with permission [160].

Contact heating is achieved by using a copper or aluminum block with high thermal conductivity. This block is poured with PDMS to create a channel, and the two layers are sealed using oxygen plasma. The copper block is then embedded in the recess of a cover sheet. The sample undergoes three stages of heating using an external heater, and the temperature is adjusted using a heating block. Nucleic acid amplification is achieved through this process. Additionally, a heating module, membrane heater, and fan-forced air are used for heating and cooling purposes. Qiu et al. developed a bedside diagnostic system [145] that utilizes a double-sided heater. This system ensures that the temperature of the liquid in the reaction chamber closely tracks the set point temperature with ±0.1 °C accuracy, minimizing the transition time between temperatures. This system was used for detecting mimic phage DNA. Xiang et al. designed a low-cost miniature PCR device [146] consisting of a disposable reactor chip and a miniature thermal cycler. The thermal cycler features a membrane heater for heating and a fan for rapid cooling. It enables combined detection with real-time fluorescence for specific DNA sequences. Neuzil et al. designed, fabricated, and tested a thermal cycler capable of completing a thermal cycle in just 8.5 s [147]. Lim et al. developed a portable PCR system [148] using a Pt film heater and dual cooling fans for low-power operation. They incorporated a film-based PCR chip, reducing the thermal mass and increasing heat transfer area, thereby improving the heat transfer efficiency. The thermal cycler design is shown in Figure 3A. Furthermore, heat transfer efficiency is enhanced by utilizing a sandwich structure with a double-heater chip on the upper and lower parts of the PCR chip. This system was used for detecting simulated viruses and *E. coli.*

Contact heating, which involves direct contact with the PCR reaction chamber, requires the entire system to be heated and cooled during thermal cycling. This significantly increases the heat capacity of the reaction system, limiting the rate at which thermal cycling can occur. To overcome this limitation, noncontact heating methods, such as infrared, laser, and microwave, have been employed. Chip-based quantitative real-time PCR (qRT-PCR) was implemented by R. Prakash et al. using an electrically driven DMF-based technology in combination with appropriately tailored resistive microheaters and temperature sensors. The device uses in vitro synthesized viral RNA fragments to detect and quantify the presence of influenza A and C virus nucleic acids [149]. Roperd et al. have developed a completely noncontact temperature system [150] for the detection of bacteria. An infrared (IR)-sensitive pyrometer was calibrated against a thermocouple inserted into the PCR chamber and used to monitor the temperature of the glass surface above the PCR chamber during heating and cooling caused by a tungsten lamp and convective air source, respectively. Ouyang et al. first demonstrated thermal cycling in a PeT microchip on an IR-PCR system [151]. A spatial filter in the form of an aluminum foil mask was used to eliminate the undesired IR absorption of the black-toner-bonded layer and successfully amplify the mock λ phage genome fragment. Tanaka et al. used a diode laser for the photothermal temperature control of enzyme-catalyzed reactions in microchips [152]. The temperature in the microchannel could be locally increased by 5–7 °C in a short time due to the heat released by the target, demonstrating that a direct solvent heating method using infrared radiation could control the reaction in the microchannel. Easley et al. investigated fiberoptic nonintrinsic Fabry–Perot interference (EFPI) as a noncontact temperature sensor and used it for the temperature regulation of small volumes of solution on microchips for the detection of simulated phages [153]. In addition, Slyadnev et al.’s miniaturized device with the infrared laser heating of the solvent based on the photothermal effect enables the rapid and local control of enzymatic reactions on microchips under flow conditions [154]. The device operates at ultrafast heating and cooling rates of 67 and 53 °C/s, respectively, which is 30 times faster than conventional systems. Liu et al. designed a novel PCR platform [155] for human tumor virus (HPV) detection. Forty cycles were completed in 90 min. Christian Fermér et al. demonstrated a PCR reaction using focused microwave irradiation as a heat source [156]. It could be determined that continuous microwave heating did not terminate the enzymatic function of the polymerase and the results showed that the total reaction time could be reduced. Yoshimura et al. studied the application of microwave heating to RCA and noted the factors contributing to the selective heating effect of microwaves [157]. This is shown in Figure 3B. Parker et al. have developed a combination of microfluidic and laser-induced fluorescence (LIF) assays to detect RT-LAMP products [158], with the optics and hardware shown in Figure 3C. Ko et al. designed a noncontact temperature-control system for integrating conventional PCR onto an LOD [159]. Experimental results showed stable PCR amplification in a single PCR-LOD, providing a one-stop-shop Salmonella detection capability. A noncontact thermal control system using an infrared thermometer and laser module as PCR amplification is shown in Figure 3D. Seo et al. have designed a fully automated CD-ROM laboratory for cell lysis and amplification on a single DNA sample CD-ROM [160], using infrared (IR) to show the temperature distribution in the amplification chamber and a laser module to keep the temperature stable. The temperature control system is shown in Figure 3E. Although the overall reaction temperature was the same, microwave heating accelerated the isothermal amplification of DNA due to the different heating mechanisms of the microwave on the temperature of the reaction components.

### 2.2. Isothermal Amplification

Isothermal amplification is a simplified and faster alternative to PCR, as it does not require denaturation, annealing, and extension steps. The method of detection of the isothermal temperature can be done using turbidimetry [161], fluorescence detection [162,163,164], gel electrophoresis [165,166,167,168], and visual color change chromogenesis [169,170,171,172].

Loop-mediated isothermal amplification (LAMP) is a method for amplifying nucleic acids at 60–65 °C, using DNA polymerase with a high strand-substitution activity. Wang et al. developed a visual LAMP assay [173] for the detection of African swine fever virus. The device was tested using DNA or cDNA from pseudotropic AIDS virus (PRV), porcine circovirus type 2 (pcv2), classical swine fever virus (CSFV), and porcine reproductive and respiratory syndrome virus (PRRS). Chen et al. used LAMP in combination with magnesium pyrophosphate precipitation to detect EHP and found that the device had good performance [174]. 

Strand Displacement Amplification (SDA) involves two enzymes: a restriction endonuclease and a DNA polymerase with strand displacement activity. The enzymes used in this method are thermally stable and can reach reaction temperatures of around 54 °C. SDA amplification is efficient, with short reaction times and high specificity. However, some single- and double-stranded products are generated in the cycle. Gong et al. developed an SDA-assisted CRISPR-Cas12a method [175] to achieve the sensitive detection of HBV DNA. Lee et al. determined S1 nuclease activity by using exponential strand displacement amplification (eSDA) inhibition [176].

The optimal reaction temperature for recombinase polymerase amplification (RPA) is around 37 °C [177]. RPA is a fast reaction and is suitable for POCT. However, the need for longer primer lengths also limits the detection of short sequences of nucleic acids. Deng et al. used the basic RPA method for performance testing on schistosome-infected nail snails and mouse models [178]. RPA was used by Lacharoje et al. to detect feline leukemia virus (FeLV) DNA provirus [179]. Mei et al. detected the SARS-CoV-2 virus and Helicobacter pylori based on RPA at a lower temperature for 30 min [180].

Rolling-loop nucleic acid amplification (RCA) technology performs nucleic acid amplification at 30 °C or room temperature [181]. A loop-locking probe, DNA ligase, and Phi29 DNA polymerase are required. The technique allows for a single-molecule detection level and the detection of RNA without reverse transcription, but requires loop-DNA templates. 

The nucleic-acid-sequence-dependent amplification technique (NASBA) performs reactions at 42 °C using AMV reverse transcriptase, T7RNA polymutase, and nuclease H. NASBA combines the reverse transcription process directly into the amplification reaction, shortening the reaction time. However, the reaction components are more complex. For example, Ju et al. built an ultrasensitive version using NASBA integration [182], with the successful detection of respiratory syncytial virus A genomic RNA (GRNA).

Some other isothermal amplification techniques, such as cross-primed amplification (CPA), use Bst DNA polymerase with strand-substitution properties and betaine to perform the reaction at around 63 °C. This technique requires fewer enzymes, but the primers are more complex. The decapping-enzyme-dependent amplification (HDA) technique utilizes decapping enzymes, single-stranded DNA binding proteins, etc. Compared to other thermostatic amplification techniques, this technique resembles a thermostatic version of the PCR. A comparison of the various aspects of isothermal amplification is shown in Table 2.

## 3. Extraction, Amplification, and Detection Fully Automated, Fully Closed Detection

Nucleic acid testing encompasses semiautomated equipment for amplification and detection, as well as fully automated equipment for extraction, amplification, and detection. Fully automated testing equipment must be free from contamination and possess speedy detection capabilities [183,184,185]. Portable fully automated nucleic acid testing devices can be employed in hospitals, customs facilities, community clinics, and other locations where technicians do not require extensive specialization but possess basic knowledge to operate the device using the accompanying manual. In remote areas with limited medical resources, portable fully automated nucleic acid testing equipment can be utilized to reach every household due to its compact size. The extraction, amplification, and detection processes are entirely automated and enclosed, incorporating cassettes and microfluidic chips.

### 3.1. Microfluidic Chips

For microfluidic chips [186,187], the functions of the entire laboratory are completely integrated on a micron-sized chip, where external forces pass through tiny and long channels to complete the extraction, amplification, and detection of nucleic acids [188,189,190,191]. In order to move the sample through the intended space, an external force is required to drive it [192,193,194]. The drives used include centrifugal drives, electromagnetic drives, external drives (syringes), capillary drives, and other drivers [195,196,197]. Due to the small volume of sample to be tested using the microfluidic chip, the testing speed is already fast and the entire process can be made even faster by designing a suitable microfluidic path [198,199,200]. 

**Figure 4:**
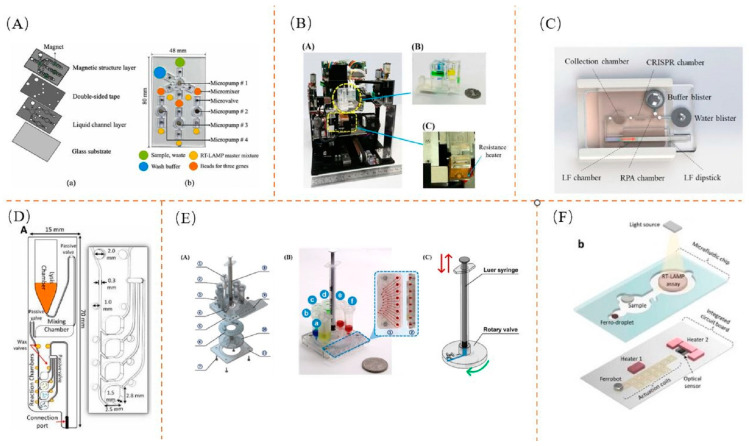
Microfluidic chips: (**A**) Microfluidic chip structure. Reproduced with permission [201]; (**B**) Device structure distribution. Reproduced with permission [130]; (**C**) Overall device structure. Reproduced with permission [202]; (**D**) Microfluidic chip structure. Reproduced with permission [89]; (**E**) Microfluidic chip combined with cassette. Reproduced with permission [204]; (**F**) Microfluidic chip assay structure. Reproduced with permission [205].

A device capable of performing lysis, extraction, amplification, and detection in a single unit using an electromagnetically driven microfluidic chip was designed by Tsai et al. [201]. This system utilizes electromagnets to control the movement of magnets, micropumps, and microvalves, facilitating the transfer of samples. The device has been designed for the detection of simian Severe Acute Respiratory Syndrome Coronavirus 2 (SAS-CoV-2). The operation begins with the first solenoid being electromagnetically driven to allow the sample to enter, followed by opening of the second solenoid to create suction with the micropump, enabling a rapid sample flow into the microchannel. Subsequently, the sample enters the chamber containing the lysis buffer to be lysed and heated to 95 °C using a thermoelectric cooler. Next, a third electromagnet is activated to divide the sample equally into three portions, each directed to a chamber containing magnetic beads for nucleic acid extraction. This process occurs at a temperature of 45 °C for 10 min. The extracted nucleic acid is collected using an electromagnet and delivered to the chamber containing the RT-LAMP solution for amplification at 60 °C. Finally, a fluorescence detection allows for quantitative analysis. To improve the experiment accuracy, positive and negative controls are included in the microarray. Accurate temperature control is necessary for the lysis, extraction, and mixing steps. Therefore, a temperature control module is placed underneath the microfluidic chip to achieve precise temperature control in the microchannel. Figure 4A illustrates the design of the microfluidic chip used in this system, which features a removable magnetic structure layer that can be reused through activation with an electromagnet. Voltage optimization was carried out to determine the most effective voltage level for electromagnetic actuation. It was found that applying a voltage of 10V delivered the best results. To enhance the sensitivity and specificity of the system, a comparative study using different primers was conducted. The results indicated that the LAMP with two or more primers demonstrated better specificity than the PCR. Additionally, successful detection of viral RNA was achieved by diluting samples at different multiplications. A microfluidic system for rapid nucleic acid analysis based on real-time convective PCR was developed by Xu et al. [130]. The system incorporates a microfluidic chip that efficiently extracts nucleic acids while utilizing convective PCR for rapid nucleic acid amplification. The microfluidic chip integrates a prestorage chamber, lysis and washing chamber, elution chamber, and waste chamber, as depicted in Figure 4B. Amplification reactions are performed using capillaries. This system has the capability to detect a wide range of viruses and necessitates the utilization of multiple handheld real-time CPCR devices. The microfluidic system successfully detects simulated influenza A (H1N1) virus concentrations as low as 1.0 TCID_50_/mL.

Li et al. developed a CRISPR-based microfluidic chip system for nucleic acid detection [202], which integrates isothermal amplification, lysis, and sidestream detection into one system to achieve contamination-free, visualized detection throughout the process, as well as the successful detection of clinical samples. In general, sidestream detection alone does not guarantee a contamination-free process, as the sample is exposed to air when added to the spiked wells and the purity of the sample cannot be guaranteed. This system, however, has a 3D-printed housing that envelops the entire reaction, so that the reagents of the reaction do not come into contact with the outside world, ensuring no contamination. The process uses CRISPR reagents lyophilized in a CRISPR reaction chamber, with the desired solution pre-existing in an air bubble, which is pressed to bring the sample into the reaction chamber. A constant temperature is required during the amplification period and the appropriate heat is provided by a hand-warming bag, eliminating the need for complex heaters. The RT-RPA mixture is then added to the reaction chamber and the solution in the RPA reaction chamber is transferred to the CRISPR reaction chamber by pressing on the bubble with the finger through the hand warmer for a period of time, allowing the lyophilized reagent in this chamber to hydrate and activate the CRISPR-Cas12a nonspecific probe, followed by a period of heating to transfer the buffer from the buffer bubble to the CRISPR solution in the reaction chamber. Finally, the mixed solution is touched to the end of the sidestream assay and the result is finally displayed in the test strip. The design structure is shown in Figure 4C. The optimal temperature of the enzyme used in the reaction chamber needed to be determined when using the hand warmer as a heater, so the appropriate temperature was selected by amplification at different temperatures and the hand-warmer-powered heater was found to be suitable for the system. To verify the clinical usefulness of the system, 17 positive and 7 negative samples were selected and the results of the assay were compared to the fluorescence images, and the results were found to be consistent with the fluorescence signal of the PT-PCR fluorescence images, indicating that the system also has good clinical diagnostic potential.

Malic et al. designed an integrated sample-to-answer system that allows for the detection of SARS-CoV-2 from NP swabs with a high sensitivity in an RT-LAMP-based manner [203]. This automated detection system is able to differentiate between positive or negative samples within 60 min with 100% agreement with the results obtained by the RT-qPCR. The system uses a pneumatically driven centrifugation platform to automate the lysis, extraction of nucleic acids, and isothermal amplification of samples on the slides. This isothermal amplification is combined with a colorimetric assay for real-time visualization without the need for complex detection equipment. The integrated microfluidic chip is set up in an enclosed space to minimize possible external contamination. The platform is equipped with a pump and pressure system to control sample entry and exit, and the centrifugal rotating platform has a heating module to complete the required heating and cooling. The designed microfluidic chip was used to validate the system on clinical patients. Seven positive and three negative patient samples were extracted and placed on the chip for testing, and a comparison of the resulting images revealed a strong correlation between the linear images. As the system uses lyophilized beads, which can be used when no relevant equipment under cryopreservation is available, it was found that the use of the RT-LAMP-lyophilized beads was successful in detecting positive samples and was perfectly compatible with field testing, allowing nucleic acid detection without the need for specialist personnel. Nguyen et al. designed an advanced IoT-based POC device [89], where liquid samples are After addition to the integrated microfluidic chip, a series of sample lysis, nucleic acid amplification, and fluorescence signals are automatically monitored in real time, and the results are transmitted to a mobile phone for display. The microfluidic chip architecture is shown in Figure 4D. Cross-contamination tests, detection limit tests, and clinical tests on the device demonstrate the practicality of the platform in the field. Four clinical respiratory virus samples (SAR-CoV-2, influenza A, antiretroviral A, and antiretroviral B) were obtained from four patients and tested on them.

The microfluidic chip can also be combined with a cassette to make the process fully automated and further improve the speed of detection. A combined chip and cassette device was designed by Li et al. that uses the RT-LAMP for nucleic acid amplification and multiplex detection by placing several primers on the chip [204]. The cassette consists of six chambers in which the solvent for nucleic acid extraction by magnetic beads, including lysate, binding solution, first and second wash solution, eluent, and RT-LAMP solution, is placed. The nucleic acid extraction and amplification reactions with the RT-LAMP solution are completed by combining a syringe with a rotary valve into the corresponding chamber, and the mixture is finally injected into the chip to complete the multiplex assay. The structure is shown in Figure 4E. In order to optimize the extraction time, each part of the extraction process is optimized to speed up the detection of nucleic acids. To improve the specificity of the overall system, each of the eight primers was chosen to be placed in the microarray, and the fluorescence curve revealed that the N gene took less time to amplify to detectable levels than the ORF1ab gene. Nucleic acid testing using this analyzer to simulate new coronaviruses can be automated in less than 80 min.

An automated NAAT-based test platform was created by Lin et al. [205]. Figure 4F shows the structure of the microfluidic chip assay. Droplet transfer, aliquoting, combining, mixing, and heating were performed by using a swarm of individually addressable millimeter-sized magnets as mobile robotic agents. Using this automated technique to detect the SARS-CoV-2 virus in clinical samples, the test results were fully consistent with those obtained from the off-chip. You-Ru Jhou developed an integrated microfluidic platform [206] for the detection and quantification of SARS-CoV-2 using RT-LAMP technology. The device automated virus lysis, RNA extraction, RT LAMP, and real-time detection. The microfluidic chip is divided into five parallel microchambers for the simultaneous detection of three genes of the new coronavirus, a positive control and a negative control. The specificity and sensitivity analysis of the device revealed by agarose gel electrophoresis that only the genes of the SARS-CoV-2 virus could be successfully amplified. The E, N, and RdRp genes of SARS-CoV-2 could be rapidly detected within 90 min for all three genes. The limit of detection for each gene was determined using three samples including synthetic RNA, inactive virus, and RNA extracted from a clinical sample infected with SAS-COV-2.

### 3.2. Cassettes

**Figure 5:**
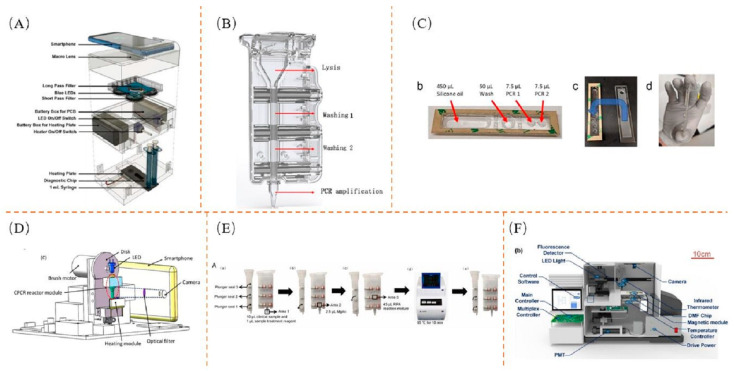
Cassettes: (**A**) Schematic diagram of the system structure. Reproduced with permission [207]; (**B**) Distribution of the various chambers in the cartridge. Reproduced with permission [211]; (**C**) Cartridge design process. Reproduced with permission [88]; (**D**) Schematic diagram of the cartridge. Reproduced with permission [214]; (**E**) Operational flow for cartridge use. Reproduced with permission [177]; (**F**) Distribution of the various functions of the cartridge. Reproduced with permission [216].

In addition to the microfluidic chip, another integrated device is the cartridge, which is more widely used because it can be used alone, but is more commonly combined with the chip [95]. Ganguli et al. designed a system that uses a cartridge based on RT-LAMP amplification in combination with a mobile phone for detection [207], which is suitable for use at the sample collection site. The collected swab is placed into a viral transport medium (VTM), the sample with the VTM is lysed, the lysed sample and RT-LAMP reagent are loaded into separate syringes of different volumes and connected to a microfluidic chip, and the fluorescence signal generated during amplification is monitored in real time via a smartphone at 65 °C. A schematic diagram of the device is shown in Figure 5A. The RT-LAMP reagent and sample need to be manually injected through the syringe into the microchannel during sample detection. The POC system was tested using 10 clinical samples and was able to detect SARS-CoV-2 from these clinical samples by differentiating positive samples from negative samples after 30 min. Infection with foodborne pathogens can have a number of undesirable consequences [208,209]; therefore, Liu et al. devised a LAMP assay that combines staining and image-processing to detect color changes in LM by smartphone, thus providing a new idea for the simple and sensitive detection of this type of pathogen [210].

Li et al. combined the qRT-PCR assay with an automated integration system for nucleic acid extraction and amplification, resulting in an automated integrated gene detection system for SARS-CoV-2 [211] in which the cartridge consisted of a lysis zone, wash zone 1, wash zone 2, and PCR amplification zone; finally, the amplification curve is displayed on the screen in real time. This is shown in Figure 5B. The detection system is a dual channel: a FAM-labeled coronavirus detection probe and a ROX-labeled internal reference detection probe. Clinical performances of the AIGS assay were assessed in 266 suspected COVID-19 clinical respiratory tract samples tested in parallel with a commercial kit. Trick et al. developed a portable magnetic fluidic cartridge platform for automated polymerase chain reaction testing in <30 min [88] for the identification of variant mutations or screening for influenza A, B, and SARS-CoV-2 in clinical samples. Magnetic beads are used to capture, purify, and transfer analytes for amplification or detection purposes through the movement of discrete reagent droplets. The design in the cartridge is as follows: With the spacer strip in place, silicone oil, wash buffer, and PCR buffer are dispensed directly into the thermoformed wells. The top layer has laser-patterned Teflon tape and laser-cut sample ports. The cartridge design process is shown in Figure 5C. The device uses a small heater, and therefore greatly reduces the energy requirement for rapid thermal cycling. Nguyen et al. present the first detailed fabrication and clinical validation of an amplified response on isothermal polymer cartridges for the rapid, in situ detection of SARS-CoV-2 using the real-time reverse transcription conversion of a point-of-care device [212]. The robustness of the system was confirmed by the analysis of 398 clinical samples initially examined in two Danish hospitals.

Yang et al. developed a portable microfluidic-based integrated assay analysis system for the detection of SARS-CoV-2 directly from saliva samples [213], where saliva cartridges are independent and evaluated using multiplex real-time quantitative polymerase chain reaction with different primer sets and mock SARS-CoV-2 internal controls. Miao et al. designed a capillary tube with FTA membranes to facilitate CPCR [214]. The lysed sample or wash buffer is centrifuged through a membrane filter and the capillary is heated for amplification using a removable resistance heater, while the reaction fluorescence signal is monitored in situ using a smartphone camera. The overall construction of one of the cartridges is shown in Figure 5D. The results show that the influenza A virus can be successfully detected within 45 min at reasonable concentrations.

Fang et al. developed a two-channel temperature controller for a portable nucleic acid detection system [183], based on the principle of nucleic acid detection by magnetic nanoparticles, which can operate in different modes, including high-precision heating control for nucleic acid extraction, fast thermal cycling control for PCR, and adjustable constant heating/cooling control. In order to test the performance of the device, a comparison is made by means of water and mineral oil, where the mineral oil prevents the evaporation of water due to high temperatures and where the actual control does not allow the direct measurement of the liquid temperature. Therefore, a linear fit was made to the relationship between the set temperature and the liquid temperature in order to achieve an accurate target value for the liquid temperature. The rapid detection of C. difficile can be achieved with this device. Tang et al. developed a rapid integrated RPA (I-RPA) system to detect nucleic acids in clinical samples [177]. This device has successfully detected SARS-CoV-2. The cartridge operation flow is shown in Figure 5E. The system does not require an additional nucleic acid extraction process and takes approximately 30 min for the sample to enter and exit.

Yoo et al. developed a nucleic-acid-based diagnostic tool to directly detect dengue virus in whole blood [215] using a combination of microbead-assisted and LAMP. The entire assay process includes sample loading, agitation, heating, and LAMP amplification. The corresponding cassette is divided into three sections, including a microfluidic channel section, a sample preparation chamber section, and a LAMP chamber section. In comparison with commercial instruments, the device was found to be successful in detecting dengue virus at lower concentrations. This method can also be applied directly to the detection of Gram-positive/negative bacteria, influenza A, etc. An all-in-one nucleic acid detection system based on DMF technology was designed by Hu et al. [216], which integrates automated nucleic acid extraction, rapid nucleic acid amplification, and real-time amplification product detection in one system. The architecture of the all-in-one system is shown in Figure 5F. Real-time LAMP reactions on discovery tablets from a selection of positive standards from commercial kits yielded almost identical test results to those of commercial machines. The successful detection of SARS-CoV-2 and *E. coli.* is mimicked.

The fully automated and enclosed nature of the extraction, amplification, and detection processes significantly reduces testing time and improves testing efficiency. This automation eliminates the need for specialized personnel to perform the assay, enabling nonprofessionals to carry out the testing procedures. Nonlaboratory testing is particularly susceptible to contamination, and to mitigate this risk, the use of fully enclosed environments, such as microfluidic chips or cassettes, becomes essential for achieving ultrafast, automated testing. In the future, locations such as community clinics, customs facilities, hospitals, and remote areas will be able to conduct rapid, portable, and fully automated nucleic acid testing. This will alleviate the burden on healthcare professionals and reduce waiting times for individuals undergoing testing.

## 4. Commercial Nucleic Acid Testing Equipment

Commercial nucleic acid testing devices for detection [217,218,219,220].Such as the instrument-free, disposable PCR testing kit developed by Visby Medical Inc, which allows for on-demand testing with rapid results [221]. Although currently authorized for laboratory testing, their device simplifies the testing process by collecting a sample and placing it in the sample port with the push of three buttons. The sample is sealed upon entering the inlet port to prevent cross-contamination. While the platform is simple to operate, it is still primarily used in laboratory environments. Future improvements are expected to further reduce the testing time and facilitate field testing. Mesa Biotech has developed the New Crown assay, which utilizes an asymmetric PCR to generate single-stranded products for hybridization with nucleic acids [222]. The test is automated, involving placing the collected sample into a self-contained tube and transferring it to the test card via a dropper. After a 30 min waiting period, test results are obtained. The entire process is enclosed, and the device has received emergency-use authorization. The system features a reusable base and disposable test cards, reducing the risk of cross-contamination. However, home testing is not yet available. Ltd. has developed an integrated field-level nucleic acid extraction and quantitative PCR system that uses ultrasonic vibration and magnetic bead adsorption for nucleic acid extraction. The collected swab is folded and added to the spiking hole of the chip, the chip is pressed to press the reagent into the tube, the instrument is started, and the fluorescence test results are automatically obtained after approximately 40 min. Currently in the development stage, this system not only offers ease of operation, but also enables the transmission of test results via wireless communication modules such as Bluetooth, enhancing the accessibility and understanding of the nucleic acid test results.

MicroGEM US Inc, a UK molecular biology company, performs saliva sampling coupled with a mobile PCR cart to extract nucleic acids using an enzymatic method, yielding results in 27 min [223]. Detect, Inc, a Connecticut-based health technology company, uses nasal swab sampling, a nucleic acid test strip detection technique based on the RT-LAMP method, with a detection time of 1 h. An all-in-one self-service SARS-CoV-2 nucleic acid detection kit developed by Tsinghua University using a colloidal gold test strip with an optimized lysis process and back-end amplification reagents and nested isothermal amplification (ITA) technology. The test can be completed by pushing the two pushers up and down, and the test takes 30 min. The Hong Kong Polytechnic University has developed a portable nucleic acid test based on the RT-LAMP and gold nanoparticles, with a test time of 25–40 min and results recognizable to the naked eye. Shanghai Speedy Diagnostics has developed the fully automated thermostatic nucleic acid amplification analyzer MA3000, a microfluidic-based fully automated thermostatic nucleic acid amplification analysis system with a test time of 30–45 min. Atas Genetics uses asymmetric PCR and electrochemical detection, as well as a microfluidic chip for the detection of sexually transmitted diseases, and it takes about 30 min to get the results [224]. The Cobas Liat PCR system uses PCR quantitative analysis, microfluidic (squeeze), a test tube, and a chip to detect influenza virus, Streptococcus, and Clostridium difficile; one target takes 20 min to detect influenza [225]. GeneXpert develops a PCR quantitative analysis, reagent lyophilized powder storage, microfluidic technology, and a cassette detection system for the detection of routine infectious diseases and cancer genes. Five targets take 60 min [226]. Filmarray utilizes a nested PCR and multiplex PCR, reagent lyophilized powder storage, microfluidic technology, microarrays for the early and rapid screening of sexual diseases; twenty-four targets take 60 min [227]. The above equipment utilizes PCR or isothermal amplification to speed up the detection times, with results typically obtained within 30–60 min. Cross-contamination is minimized due to its hermetic seal. The sample to be tested is placed in the test air and the start button is pressed to automatically perform the nucleic acid test and obtain the results. Currently, there are few home nucleic acid testing devices available, and some commercial instruments are not suitable for home testing. Also, the test time is still longer compared to general antigen testing, but the sensitivity of the test is still reliable.

Paper-based microfluidics combined with other isothermal amplification techniques are commonly employed for visualizing nucleic acid detection results [228]. For instance, a diagnostic card for early stage pancreatic cancer has been developed, featuring dual amplification capability on a paper chip. This card utilizes quantum dots and multicolor fluorescent labeling instead of a gold enhancement step, allowing for the accurate quantification of miRNA-21 and hepatitis C virus, while reducing the reaction time to one-third of the original time. A comparison of various commercial instruments is shown in Table 3.

## 5. Conclusions

Some common nucleic acid testing methods require bulky equipment, specialized technicians, and laboratory settings. However, in order to alleviate the burden on professionals and facilitate testing in remote and resource-limited locations, point-of-care nucleic acid testing has gained prominence. As a result, current trends in nucleic acid testing equipment are focused on improving the three main components of testing: nucleic acid extraction, amplification, and detection. Nucleic acid extraction is a crucial step that can be broadly divided into three stages: fragmentation, extraction, and purification. Advancements in these processes have led to faster and more portable tests. Some testing devices have even eliminated the need for nucleic acid extraction altogether. In order to meet the high demand for testing and expedite results, it is essential to choose the most suitable methods for each step. There are various approaches available, and the key lies in selecting the right method that ensures speed and efficiency. Nucleic acid amplification techniques, including variable temperature amplification and isothermal amplification, require precise temperature control. Improving the rate of temperature increase is crucial for overall speed, while considering the temperature variations that can impact the functionality of the amplification enzymes. Maintaining an optimal temperature range ensures successful amplification. In terms of testing methods, visual or instrument-based results are sought for rapid point-of-care testing. Steps involved in nucleic acid testing often require specialized expertise, but for broader accessibility, automated operation is more desirable. Cartridges and chips simplify the process by allowing the collected sample to be placed into the test port, and results can be obtained by pressing a button after a specific waiting period. Visual inspection determines negative or positive results. Automated testing significantly reduces the testing time, and the enclosed space of the system minimizes contamination and interference. While larger sample sizes are typically required for reliable testing, using smaller sample sizes can expedite the process. Microfluidic channels are employed for detection, where the sample passes through a small and elongated channel, undergoes nucleic acid extraction, and then undergoes amplification under different temperature control. Integrating microfluidic chips into the overall nucleic acid assay system makes the device more compact and portable, utilizing smaller sample volumes for qualitative or quantitative analysis.

Currently, there is room for improvement in some assay equipment used for nucleic acid testing. Although automated testing systems have made significant progress in detecting disease-causing viruses, there are still instances of false-positive results, and efforts are underway to enhance the accuracy of these devices in the future. Another area that requires attention is the time taken to obtain test results, which needs improvement to achieve faster testing. There is also a need to integrate testing modules and make the devices more compact and efficient. Furthermore, the development of home-testing devices for nucleic acid testing is relatively limited, and there is a demand for more user-friendly devices that can enable rapid self-testing at home, catering to specific needs. Additionally, CRISPR-based technology shows promise in detecting small amounts of RNA without the need for amplification. However, there are currently few commercially available instruments based on CRISPR technology, and further research could be conducted to explore the potential of this detection technology. In order to address these challenges, the focus of future developments will be on creating simple, rapid, noncontaminating, and point-of-care devices. These advancements aim to provide improved testing environments and systems for the early detection of pathogenic viruses.

## Figures and Tables

**Table 1:** The most commonly studied pathogens for nucleic acid testing.

Pathogens	Genetic Material	Principle of Detection	Detection Time(min)	Detection Method	Authenticity of the Sample	References
MERS	RNA	RT-RPA	>30	Immunochromatography	Simulated samples	[92]
EBOV	RNA	RT-LAMP	40	Immunochromatography	Simulated samples	[93]
SARS-CoV-2	RNA	RT-LAMP-CRISPR	45	Fluorescence detection	Clinical samples	[94]
HIV	RNA	RT-LAMP	60	Fluorescence detection	Simulated samples	[95]
H1N1	RNA	RPA	Amplification time of 25	Fluorescence detection	Clinical samples	[96]
ZIKV	RNA	RT-LAMP	32	Fluorescence detection	Simulated samples	[97]
HCV	RNA	RT-RAA	30	Immunochromatography	Clinical samples	[98]
NV	RNA	Biosensing	30	Colorimetric detection	Simulated samples	[99]
HBV	DNA	CRISPR/Cas12a	50	Raman scattering	Simulated samples	[100]
Pneumococcus	DNA	RT-PCR	45	Electrophoretic detection	Clinical samples	[101]
Salmonella	DNA	LAMP	40	Colorimetric detection	Simulated samples	[102]

**Table 2:** Comparison of the characteristics of common isothermal amplifications.

Classification	Temperature(°C)	Number of Primers	Number of Enzymes	Advantages	Disadvantages
LAMP	60–65	4–6	1	Short reaction time, higher specificity	Primer complexity
RPA	30–42	4	3	Rapid reaction time, 5–30 min to reach detection level	Long primers and probes
RCA	37	1 or more	1	High efficiency, single molecule detection levels can be achieved	Requires circular DNA template
SDA	37	4	2	High amplification efficiency, short reaction time, high specificity, no special equipment required	Heterogeneous products
CPA	63	5–6	1	Fewer enzymes involved, high sensitivity and specificity	Primers are more complex
HAD	37	2	2	Simple primers	Suitable for amplifying short sequences
NASBA	42	2	3	Reverse transcription is incorporated into the amplification, reducing reaction time	Reaction components are more complex

**Table 3:** Nucleic acid testing equipment for commercial instruments.

Company	Time (min)	Amplification Method	Study Content	Automation
Visby Medical Inc.	≥30	Continuous flow PCR	for detection of neocoronavirus	Fully automated
Mesa Biotech	≥30	Asymmetric PCR	for detection of neocoronavirus	Fully automated
Beijing Zhongke Biotech	≥40	PCR	for detection of neocoronavirus	Fully automated
MicroGEM US Inc.	≥27	PCR	for detection of neocoronavirus	Fully automated
Detect, Inc.	≥60	RT-LAMP	for detection of neocoronavirus	Fully automated
Tsinghua University	≥30	data ITA	for detection of neocoronavirus	Fully automated
Hong Kong Polytechnic University	25–40	RT-LAMP	for detection of neocoronavirus	Fully automated
Atas Genetics io	30	Asymmetric PCR	for STI detection	Fully automated
GeneXpert	targets/60 min	PCR	for routine infectious diseases	Fully automated
Filmarray	24 targets/60 min	Nested PCR + multiplex PCR	for early stage sexually transmitted diseases	Fully automated
Cobas Liat PCR system	1 target/20 min	PCR	for influenza virus	Fully automated

## Data Availability

Not applicable.
